# The Complete Mitochondrial Genome of *Torix tukubana* (Annelida: Hirudinea: Glossiphoniidae)

**DOI:** 10.3390/genes14020388

**Published:** 2023-02-01

**Authors:** Xiaochen Zhu, Yingying Zhao, Hua Wei, Nan Hu, Qingbiao Hu, Yingdong Li

**Affiliations:** 1 Key Laboratory of Livestock Infectious Diseases in Northeast China, Ministry of Education, College of Animal Science and Veterinary Medicine, Shenyang Agricultural University, Shenyang 110866, China; 2 College of Science and Engineering, Flinders University, Adelaide 5024, Australia; 3 Liaoning Panjin Wetland Ecosystem National Observation and Research Station, Shenyang 110866, China

**Keywords:** mitochondrial genome, leech, *Torix tukubana*, comparative analyses, phylogenetics

## Abstract

*Torix tukubana* is a poorly understood proboscidate leech species, generally an ectoparasite on amphibian species. In this study, the complete mitochondrial genome (mitogenome) of *T. tukubana* was sequenced using next-generation sequencing (NGS), and the essential characteristics, gene arrangement, and phylogenetic relationship were analyzed. The results showed that the *T. tukubana* mitogenome was 14,814 bp in length, consisting of 13 protein-coding genes (PCGs), 22 *tRNAs*, 2 *rRNAs*, and 1 control region (CR). The mitogenome composition presented a strong A + T bias (73.6%). All *tRNAs* had the typical clover structure except the *trnS1* (TCT), whose dihydrouridine (DHU) arm was short, having only one complementary base pair. Additionally, 8 gene order patterns were identified among 25 known Hirudinea species, and *T. tukubana* was identical to the Hirudinea ground pattern. A phylogenetic analysis based on 13 PCGs indicated that all the studied species clustered into three main clades. The relationships among Hirudinea species were basically consistent with their gene arrangement results, but different from their morphological taxonomy. *T. tukubana* was in the monophyletic group of Glossiphoniidae, a finding consistent with previous research. Our results provided the essential characteristics of the *T. tukubana* mitogenome. As the first complete mitogenome of *Torix*, it could offer valuable information for a systematic understanding of the Hirudinea species.

## 1. Introduction

Hirudinea, or leeches, are clitellate annelids and are widely distributed in various ecosystems (e.g., aquatic and moist terrestrial) on all continents [1]. Leeches are usually ectoparasites or predators and play an integral role in the freshwater system [2]. The phylogenetic relationship between Hirudinea subclasses has always been disputable in terms of their morphological taxonomy and molecular phylogeny. Molecular technology therefore seems necessary for simplified and accurate identification due to the morphological intraspecific variability exhibited among Hirudinea species [3]. Because of their rapid mutation rate and lack of recombination, mitochondrial DNAs have been extensively applied in population genetics and intra- or inter-specific phylogenetic studies [4,5]. The comparison of complete mitochondrial genomes (mitogenomes) has become increasingly popular in phylogenetic relationship analyses [6,7,8] because complete mitogenomes are more informative than single genes [9]. Additionally, complete mitogenomes can reveal some genome-level characteristics, such as gene rearrangement [10,11,12], which could provide more information for understanding the phylogenetic relationships between species [13,14]. Although they are of significant help in phylogenetic and population genetics, only a few complete mitogenomes of the approximately 680 Hirudinea species have been determined so far [6,15,16,17].

*Torix tukubana* (Annelida: Hirudinea: Glossiphoniidae) is a poorly understood proboscidate leech species, generally an ectoparasite on amphibian species [3,18]. According to previous records, *T. tukubana* inhabit mountain streams in Japan, and their host amphibians consist of only three Japanese frogs, *Rana japonica*, *Rana tagoi*, and *Onychodactylus japonicas* [19]. However, individual *T. tukubana* (morphological identification) specimens have recently been found to parasitize on the body surface of *Rana dybowskii* (which has great medicinal and economic value) in Dandong, Northern China. Because *T. tukubana* has been considered endemic to Japan, we amplified the specimen’s *cox1* gene and conducted nucleotide alignment to confirm the accuracy of the morphological identification. We found that the *cox1* gene of the specimen was identical to that of *T. tukubana* (GenBank: LC538265). Like other Glossiphoniidae parasites, *T. tukubana* can cause a range of impairments in their hosts, including tissue damage and weight loss [20]. In addition, infestation of *T. tukubana* is also associated with Rickettsia infection [20]. The outbreak of *T. tukubana* is doubtlessly a potential menace to the aquaculture industry and the wild resource of *R. dybowskii*.

In previous studies, the understanding of phylogenetic relationships among leech species, including *T. tukubana*, were mainly based on short fragments, such as mitochondrial (12S *rRNA*, *cox1*, *nad1*) and nuclear ribosomal internal transcribed spacer (*ITS*) gene sequences [17,21,22,23,24]. Therefore, the complete mitogenome might provide new insight into the phylogenetic relationships between leech species. Currently, the research on *T. tukubana* consists mainly of morphological descriptions, with few molecular studies based on the mitochondrial *cox1* gene [18]. There has been no report of the complete mitogenome of *T. tukubana*. In this study, the complete mitogenome of *T. tukubana* was obtained utilizing next-generation sequencing (NGS). As this is the first mitogenome in the genus *Torix*, the results will enrich the Hirudinea molecular database. Furthermore, the phylogenetic relationships among 25 complete Hirudinea mitogenomes were analyzed, the results of this analysis are of great importance to the future study of their phylogenetic relationship and will improve our understanding of the genetic biodiversity of Hirudinea.

## 2. Materials and Methods

### 2.1. Sample Collection and DNA Extraction

Twenty-six *T. tukubana* specimens were collected from the body surface of an *R. dybowskii*, which inhabited the mountain of Dandong City (40.884° N; 124.762° E), Liaoning Province, China, in October 2020. The body sizes were from 0.56 cm to 0.89 cm (0.765 cm ± 0.034 SD). The *T. tukubana* specimens were identified based on the external morphological features described by Kambayashi et al. [19] and *cox1* gene alignment.

Before DNA isolation, the specimen was immediately stored in liquid nitrogen. A TIANamp Marine Animals DNA Kit (Tiangen, China) was used to extract the total genomic DNA from one *T. tukubana*, and the degree of concentration and purity of the DNA was evaluated using a Thermo Scientific NanoDrop 2000. The integrity was evaluated using an Agilent 2100 Bioanalyzer and electrophoresis on a 1% agarose gel.

### 2.2. Genome Assembly and Annotation

Briefly, after the genomic DNA was randomly broken into fragments using a Covairs ultrasonic breaker, a PE400 genomic DNA library was constructed based on the Whole Genome Shotgun (WGS) strategy and sequenced utilizing an Illumina NovaSeq 6000 instrument (Illumina, USA). More than 2.58 Gb of raw reads were obtained, and at least 1.67 Gb of clean reads were obtained from the raw reads after removing adaptor sequences using AdapterRemoval v2 [25] and filtering low-quality sequences using SOAPdenovo2 [26]. The obtained clean data were assembled using A5-miseq v20150522 [27] and SPAdesv3.9.0 [28] to construct contigs and scaffolds. A colinear analysis for splicing sequences was conducted using mummer v3.1 [29] and the complete mitogenome sequence was then revised and verified using pilon v1.18 [30]. All these procedures were performed by Shanghai Personal Biotechnology Co., Ltd., China.

The locations of protein-coding genes (PCGs) and *rRNA* genes were preliminarily predicted using GeSeq [31] and MITOS [32], and the precise locations were confirmed using related Hirudinea mitogenomes from GenBank. The initiation and termination codons were identified using ORF finder and Blastn of NCBI, according to their alignment with other related species, and the rearrangements of mitochondrial genes in the Hirudinea species were examined simply by observation. MITOS [32] was used to predict and annotate the locations and secondary structures of *tRNA* genes, and these were then visualized using the online software TBI-forna (http://rna.tbi.univie.ac.at/forna/, accessed on 4 January 2022). The nucleotide composition and the relative synonymous codon usage (RSCU) were determined using MEGA 7 [33].

AT skew = (A − T)/(A + T) and GC skew = (G − C)/(G + C) were analyzed to describe base composition [34]. A graphical diagram of the complete mitogenome was drawn using the online mitochondrial visualization tool mtviz (http://pacosy.informatik.uni-leipzig.de/mtviz/mtviz, accessed on 4 January 2022). Finally, the complete mitochondrial DNA sequence was uploaded to the GenBank database with accession number OL779256.

### 2.3. Phylogenetic Analysis

The mitogenome sequences of 24 Hirudinea species other than *T. tukubana* were obtained from GenBank for phylogenetic analysis (Table 1). The nucleotide sequences of 13 PCGs were aligned using the online software Clustal Omega (https://www.ebi.ac.uk/Tools/msa/clustalo/, accessed on 4 January 2022). Moreover, the poorly aligned regions and divergent sites were removed utilizing the online software Gblocks (http://molevol.cmima.csic.es/castresana/Gblocks_server.html, accessed on 4 January 2022) [35]. The online software IQ-TREE (http://iqtree.cibiv.univie.ac.at/, accessed on 6 January 2022) [36] was used to build a maximum likelihood (ML) phylogenetic tree which was visualized using iTOL (https://itol.embl.de, accessed on 6 January 2022) [37]. In addition, the mitogenomes of *Duplodicodrilus schmardae* (KT429015.1) and *Tubifex tubifex* (MW690579.1) were employed as an outgroup taxon.

## 3. Results and Discussion

### 3.1. Genome Composition

**Figure 1:**
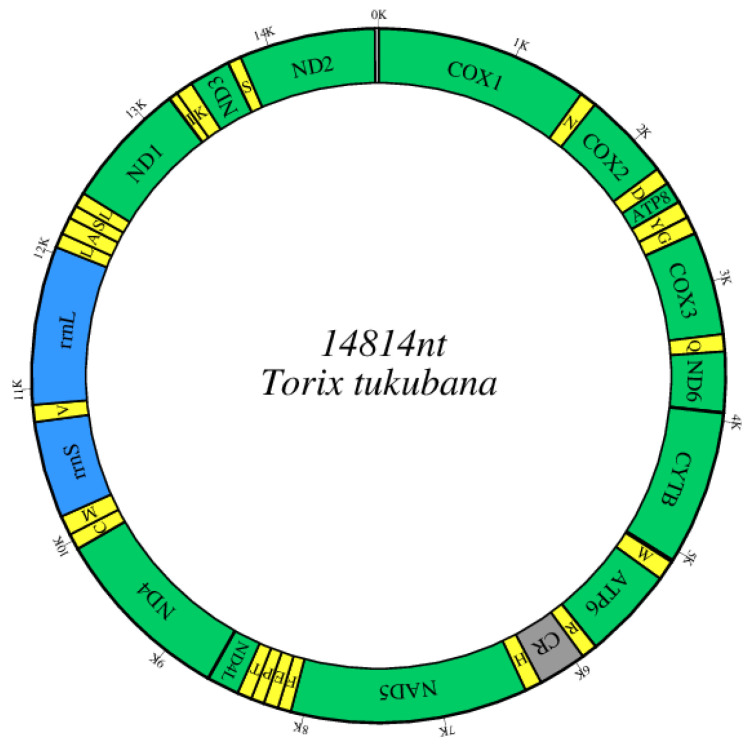
Graphical map of the mitogenome of *T. tukubana* (generated by mtviz). PCGs and *rRNA* genes are shown using standard abbreviations. PCGs are green, *tRNAs* are yellow, *rRNAs* are blue, and the CR is shown in gray. Genes for *tRNAs* are abbreviated using a single letter (N = Asn; D = Asp; Y = Tyr; G = Gly; Q = Gln; W = Trp; R = Arg; H = His; F = Phe; E = Glu; P = Pro; T = Thr; C = Cys; M = Met; V = Val; L = Leu; A = Ala; S = Ser; I = Ile; K = Lys). CR = control region. The outside line and inside line indicate the heavy chain and the light chain, respectively. The bold line represents the transcribed strand, and all genes are encoded on the same strand.

After assembly, a circular mitogenome with a length of 14,814 bp was ultimately generated (Figure 1), and this was in the range of the known Hirudinea mitogenomes (14,407–16,161 bp). There were strong similarities between the whole *T. tukubana* mitogenome and those of closely related species (84.75% with *Hemiclepsis yangtzenensis*, 82.45% with *Theromyzon tessulatum*, and 78.62% with *Helobdella robusta*).

The gene content of the *T. tukubana* mitogenome, including 13 PCGs, two *rRNA* genes, 22 *tRNA* genes, and a control region (CR; Table 2 and Figure 1), was similar to that of all known Hirudinea. All 37 genes were coded on the heavy chain, as in all known Hirudinea. Like most Hirudinea mitogenomes, the mitogenome of *T. tukubana* was closely aligned, with only a small number of bases overlapping adjacent genes, indicating efficient RNA transcription and protein translation (Table 2).

The genome composition (A: 35.2%, G: 11.9%, T: 38.4%, C: 14.5%) presented a strong A + T bias (73.6%), and showed a negative AT skew (−0.043) and GC skew (−0.098). The AT skew was higher than that of most Hirudinea mitogenomes in this study, but lower than that of *Acanthobdella peledina* (−0.007), *Codonobdella* sp. IK-2021 (−0.030), *Erpobdella japonica* (−0.025), *Erpobdella* sp. JP-2021 (−0.032), *Erpobdella testacea* (−0.004), *H. yangtzenensis* (−0.034), and *Ozobranchus jantseanus* (−0.033) (Table 1). The GC skew of *T. tukubana* was lower than that of most of the other previously sequenced species (Table 1). However, different regions of the *T. tukubana* mitogenome had similar A + T contents, ranging from 73.1% (CR) to 74.5% (*tRNAs*; Table S1).

### 3.2. Protein-Coding Genes

The PCG region was 11,097 bp in length and accounted for 74.91% of the *T. tukubana* mitogenome. The size of the 13 PCGs ranged between 159 bp and 1698 bp (Table 2). Each PCG was initiated by a canonical ATN codon, and the termination codons were TAA and TAG. Of the 13 PCGs, three (*atp8*, *nad4*, and *nad3*) terminated with TAG, and the other 10 PCGs used a typical TAA termination codon (Table 2). The number of bases in the 13 PCGs followed the pattern T > A > C > G, and they showed a strong A + T bias (73.3%; Table S1). The bias of T and C was strong, and the AT skew and GC skew were −0.080 and −0.143, respectively. The *T. tukubana* mitogenome had a slightly negative AT skew, indicating a higher incidence of the T than of the A nucleotide, a feature observed in other mitogenomes where the AT skew is negative. In addition, the *T. tukubana* mitogenome had lower GC skew values than most other mitogenomes, while its AT skew values were higher (Table S1).

**Figure 2:**
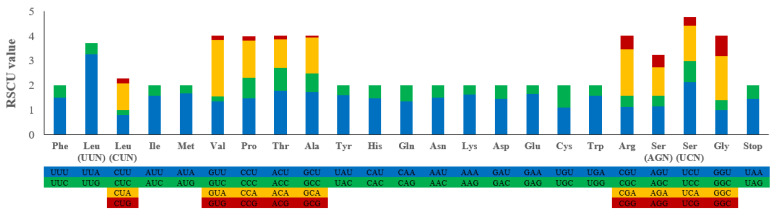
RSCU in the *T. tukubana* mitogenome. Codon families for amino acids and stop codons are shown on the x-axis and RSCU values are shown on the y-axis.

The average codon frequencies of the PCGs were calculated, and the results showed that the RSCU values of the preference codons were all greater than 1 (Figure 2). The results of this study showed that the codons of all the PCGs had a strong preference. The RSCU values of NNU and NNA (namely the codon of the third site, U or A) were mostly higher than 1, and the frequency of usage was higher, which was consistent with *Whitmania* spp. [15].

### 3.3. tRNAs, rRNAs, and CR

The *T. tukubana* mitogenome contained 22 *tRNA* genes, all located on the H strand (Figure 1, Table 2), a characteristic shared by most Hirudinea. The *tRNA* sequences ranged from 59 bp to 69 bp, with strong A + T bias (73.9%) and positive AT skew (0.034; Table S1). All of the 22 *tRNAs* showed the typical cloverleaf structure except *trnS1* (TCT), whose dihydrouridine (DHU) arm was short, having only one complementary base pair (Figure S1), a characteristic not seen in *Whitmania* spp. [15].

The *rrnS* and *rrnL* genes were located between *trnM* and *trnV* and between *trnV* and *trnL1*, and they were 735 bp and 1160 bp in length, respectively. The A+T content of this *rRNA* region was 74.5% and showed a positive AT skew (0.090) and GC skew (0.043). The CR was 402 bp, situated between *trnR* and *trnH*, and had a positive AT skew (0.130) and negative GC skew (−0.056; Table S1). The CR length of Hirudinea, ranging from 77 bp to 1670 bp, showed significant length differences [6,15,17,38]. The CR of the *T. tukubana* mitogenome was of a moderate size. That is a common phenomenon, as the CR is generally considered to have the most significant length variation in the mitogenome [10].

### 3.4. Gene Arrangement

**Figure 3:**
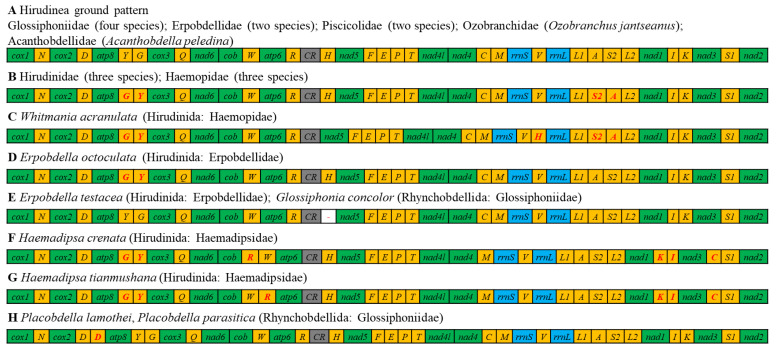
Gene order patterns of Hirudinea mitogenomes. Gene segments are not drawn to scale. All genes are encoded on the same strand. The rearrangements of *tRNAs* are shown in red.

All 37 genes of all the studied Hirudinea species, including *T. tukubana*, were transcribed from the same strand (Table 2, Figure 1), which was identical to that of all known Hirudinea species in previous studies [6,15,17]. The gene order and orientation of the 13 PCGs in *T. tukubana* were consistent with those of all previously sequenced annelids. However, *tRNAs* are generally believed to change their relative position much faster than the PCGs and *rRNAs*. The rearrangement of *tRNAs* has been identified in known annelids sequences [6]. Among all 25 known Hirudinea mitogenomes, eight different gene order patterns (A to H) have been identified (Figure 3). The gene order of the *T. tukubana* mitogenome was the same as the ground pattern (A) which was found in most of the studied Hirudinea mitogenomes. Another representative gene order pattern (B) was identified in three Hirudinidae species and three Haemopidae species. The main differences between these two patterns were translocations between *trnG* and *trnY* and *trnA* and *trnS2* (TGA). Patterns E and H were found in two species. Compared with the ground pattern (A), pattern E included a deletion of *trnH* between CR and *nad5*, while pattern H included an extra *trnD* between *cox2* and *atp8*. The other four patterns were identified in one species. In comparison with pattern B, pattern C included a relocation of *trnH*, while pattern D included relocations of *trnA* and *trnS2* (TGA). Patterns F and G included translocations of *trnR* and *trnC* and a rearrangement between *trnK* and *trnI* which differed from the other six patterns. The difference between patterns F and G was the gene order of *trnR* and *trnW*.

### 3.5. Phylogenetic Analysis

**Figure 4:**
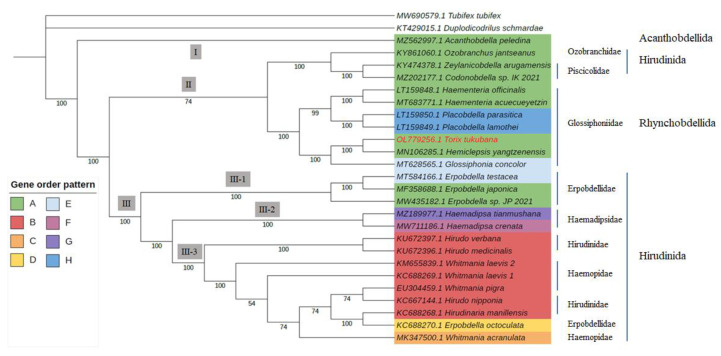
Topology derived from 13 concatenated mitochondrial PCGs from Hirudinea mitogenomes using ML analysis (generated by iTOL). The Roman numerals represent main clades. The numbers below the branches are the % bootstrap support values from the ML analysis (values below 54 are not shown).

In this study, the concatenated PCGs derived from the 25 Hirudinea mitogenomes belonging to 8 families of 14 genera were used for the phylogenetic relationship analysis (Table 1). The results of an ML analysis showed that each cluster established a phylogenetic tree with high support values (Figure 4). All species clustered into three main clades, except for the outgroups. As a primitive parasitic seawater species, *A. peledina*, which belongs to Acanthobdellida, was exclusive to one main clade (Ⅰ). Three species of Oceanobdelliformes formed a monophyletic group, and seven species of Rhynchobdellida: Glossiphoniidae, including *T. tukubana*, formed a monophyletic group, and then these groups clustered into one main clade (Ⅱ). In another main clade (Ⅲ), three Erpobdelliformes species formed a monophyletic group (Ⅲ-1), and two Haemadipsa species formed another branch (Ⅲ-2). They then clustered with the other eight species of Hirudiniformes and one of Erpobdelliformes (*Erpobdella octoculata*) in clade Ⅲ-3. The phylogenetic relationship within the Glossiphoniidae family showed that the genus *Torix* was more similar to *Hemiclepsis*, an observation which conforms with previous analyses by Kambayashi et al. [19] and Xu et al. [39].

The phylogenetic relationships within the Hirudinea class have always been subject to debate in studies of their morphological cladistics and molecular phylogeny [40]. Most of the previously published studies on phylogenetic relationships among leech species, including *T. tukubana*, were based on short barcode sequences like mitochondrial (*rrnS*, *cox1*, and *nad1*) or nuclear *ITS* genes [17,21,22,23,24]. The phylogenetic tree in this study was similar to those of previous studies using 13 PCGs or complete mitogenomes [16,17,41]. Overall, we found that Glossiphoniidae was monophyletic, but that the Erpobdellidae, Haemadipsidae, Haemopidae, and Hirudinidae families were not. For example, *E. octoculata* clustered with the Hirudiniformes species in clade Ⅲ-3, whereas the other three *Erpobdella* species formed a monophyletic branch. This result matches those of previous studies based on *cox1* sequences [15] and complete mitogenomes [41]. It is worth noting that their gene order patterns also indicated that they were probably not genetically homologous.

The phylogenetic tree in this study exhibited a certain degree of consistency in its gene order rearrangement results. As shown in Figure 3, the ground gene order (pattern A) occurred widely in clades Ⅰ, Ⅱ, and Ⅲ-1. This result confirmed that this pattern was the basic pattern among Hirudinea mitogenomes. Patterns E and H, which had a single *tRNA* deletion or addition compared with pattern A, also occurred in clades Ⅱ and Ⅲ-1. An important feature was the translocation of *trnG* and *trnY* (patterns B, C, D, F, and G), distinguishing the above clades from clades Ⅲ-2 and Ⅲ-3. Another representative pattern, B, only occurred in clade Ⅲ-3. Patterns C and D, which are similar, also existed in the same clade. As mentioned above, patterns F and G significantly differed from patterns A and B. They existed in clade Ⅲ-2, which also diverged early in clade Ⅲ. We are certain that since there exist only a tiny number of archived Hirudinea mitogenomes, more mitogenomes will be needed to reveal their phylogenetic relationships and the correlations between gene rearrangement and molecular phylogeny.

## 4. Conclusions

The first complete mitogenome of *T. tukubana* was determined in this study. All 37 genes of the *T. tukubana* mitogenome were encoded on the heavy chain, and the gene order of 13 PCGs was completely consistent with those of all known Hirudinea sequences. The *T. tukubana* mitogenome composition presented a strong A + T bias (73.6%). The 22 *tRNAs* had the typical clover structure, except *trnS1* (TCT), whose dihydrouridine (DHU) arm was short, having only one complementary base pair. All 25 known Hirudinea mitogenomes can be divided into eight gene order patterns based on the rearrangement of *tRNAs*. The main differences between these two representative patterns were rearrangements between *trnG* and *trnY* and between *trnA* and *trnS2* (TGA). The gene order of the *T. tukubana* mitogenome was identical to that of the Hirudinea ground pattern. The phylogenetic analysis based on 13 PCGs indicated that the 25 studied Hirudinea mitogenomes clustered into three main clades in a manner basically consistent with their gene rearrangement. *T. tukubana* and six other Glossiphoniidae species formed a monophyletic group. The analysis of the complete *T. tukubana* mitogenome could help us to understand this species’ basic mitogenome characteristics. It has also provided valuable molecular information for parasitological and systematic studies of Hirudinea. However, considering the tiny number of archived mitogenomes, more complete mitogenomes are essential to reveal the phylogenetic relationships within Hirudinea species.

## Figures and Tables

**Table 1:** Genomic characteristics of Hirudinea mitogenome acquired from GenBank.

Species	Accession No.	Size (bp)	Nucleotide Composition/%					AT Skew	GC Skew
			T(U)	C	A	G	A + T (U)		
*T. tukubana*	OL779256	14,814	38.4	14.5	35.2	11.9	73.6	−0.043	−0.098
*Acanthobdella peledina*	MZ562997.1	18,528	35.2	19.0	34.7	11.1	69.9	−0.007	−0.262
*Codonobdella* sp. IK-2021	MZ202177.1	14,486	38.9	12.3	36.6	12.2	75.5	−0.030	−0.004
*Erpobdella japonica*	MF358688.1	14,725	37.0	14.9	35.2	13.0	72.2	−0.025	−0.068
*Erpobdella octoculata*	KC688270.1	14,407	40.6	12.5	30.9	15.9	71.5	−0.136	0.120
*Erpobdella* sp. *JP-2021*	MW435182.1	15,469	36.0	17.4	33.8	12.8	69.8	−0.032	−0.152
*Erpobdella testacea* *	MT584166.1	14,495	36.7	14.5	36.4	12.4	73.1	−0.004	−0.078
*Glossiphonia concolor* *	MT628565.1	14,548	39.2	13.1	35.5	12.2	74.7	−0.050	−0.036
*Haemadipsa crenata*	MW711186.1	14,725	42.4	10.9	34.4	12.3	76.8	−0.104	0.060
*Haemadipsa tianmushana*	MZ189977.1	14,625	42.8	10.5	35.1	11.6	77.9	−0.099	0.050
*Haementeria acuecueyetzin*	MT683771.1	14,985	38.9	14.9	34.8	11.4	73.7	−0.056	−0.133
*Haementeria officinalis* *	LT159848.1	14,849	39.0	15.1	34.3	11.7	73.3	−0.064	−0.127
*Hemiclepsis yangtzenensis*	MN106285.1	14,984	37.7	15.0	35.2	12.1	72.9	−0.034	−0.107
*Hirudinaria manillensis*	KC688268.1	14,470	40.7	12.2	31.4	15.8	72.1	−0.129	0.129
*Hirudo medicinalis* *	KU672396.1	14,729	42.8	10.7	33.4	13.2	76.2	−0.123	0.105
*Hirudo nipponia*	KC667144.1	14,414	40.9	11.9	31.7	15.5	72.6	−0.127	0.131
*Hirudo verbana* *	KU672397.1	14,604	43.2	10.5	33.7	12.7	76.9	−0.124	0.095
*Ozobranchus jantseanus*	KY861060.1	14,864	37.4	14.9	35.0	12.6	72.4	−0.033	−0.084
*Placobdella lamothei*	LT159849.1	15,190	36.4	18.0	31.7	13.9	68.1	−0.069	−0.129
*Placobdella parasitica* *	LT159850.1	14,909	37.8	15.5	34	12.7	71.8	−0.053	−0.099
*Whitmania acranulata*	MK347500.1	14,468	40.8	12.1	30.8	16.3	71.6	−0.140	0.148
*Whitmania laevis 1*	KC688269.1	14,433	41.2	12.0	30.8	160.	72.0	−0.144	0.143
*Whitmania laevis 2*	KM655839.1	14,442	41.9	11.1	31.1	15.9	73.0	−0.148	0.178
*Whitmania pigra*	EU304459.1	14,426	41.3	11.8	30.8	16.1	72.1	−0.146	0.154
*Zeylanicobdella arugamensis*	KY474378.1	16,161	43.7	10.4	35.5	10.3	79.2	−0.104	−0.005

*: Incomplete mitogenome sequence.

**Table 2:** Annotation of *T. tukubana* mitogenome.

Gene	Position	Gene Length/bp	Start Codon	Stop Codon	Anti Codon	H/L Strand	Intergenic/Overlapping Region Length/bp
*cox1*	1–1537	1537	ATG	T--		+	
*trnN*	1538–1601	64			GTT	+	
*cox2*	1602–2279	678	ATG	TAA		+	1
*trnD*	2281–2344	64			GTC	+	
*atp8*	2345–2503	159	ATG	TAG		+	−2
*trnY*	2502–2567	66			GTA	+	−2
*trnG*	2566–2626	61			TCC	+	−3
*cox3*	2624–3404	781	ATA	T--		+	
*trnQ*	3405–3473	69			TTG	+	1
*nad6*	3475–3945	471	ATG	TAA		+	−8
*cob*	3938–5074	1137	ATG	TAA		+	16
*trnW*	5091–5154	64			TCA	+	1
*atp6*	5156–5860	705	ATG	TAA		+	−2
*trnR*	5859–5926	68			TCG	+	
*CR*	5927–6328	402					
*trnH*	6329–6392	64			GTG	+	
*nad5*	6393–8094	1702	ATG	T--		+	
*trnF*	8095–8157	63			GAA	+	
*trnE*	8158–8216	59			TTC	+	1
*trnP*	8218–8281	64			TGG	+	
*trnT*	8282–8343	62			TGT	+	
*nad4l*	8344–8637	294	ATG	TAA		+	−7
*nad4*	8631–9965	1335	ATG	TAG		+	−2
*trnC*	9964–10,027	64			GCA	+	
*trnM*	10,028–10,089	62			CAT	+	
*rrnS*	10,090–10,824	735				+	
*trnV*	10,825–10,887	63			TAC	+	
*rrnL*	10,888–12,047	1160				+	
*trnL1*	12,048–12,107	60			TAG	+	
*trnA*	12,108–12,167	60			TGC	+	−1
*trnS2*	12,167–12,233	67			TGA	+	
*trnL2*	12,234–12,294	61			TAA	+	
*nad1*	12,295–13,233	939	ATG	TAA		+	5
*trnI*	13,239–13,300	62			GAT	+	
*trnK*	13,301–13,365	65			TTT	+	
*nad3*	13,366–13,719	354	ATG	TAG		+	−3
*trnS1*	13,717–13,782	66			TCT	+	1
*nad2*	13,784–14,788	1005	ATT	TAA		+	25

## Data Availability

All data sets used in this study were provided by GenBank.

## Supplementary Materials

The following supporting information can be downloaded at: https://www.mdpi.com/article/10.3390/genes14020388/s1, Figure S1: Predicted secondary structures of the 22 *tRNA* genes of the *T. tukubana* mitogenome; Table S1: Composition and skewness in PCGs, *tRNAs*, *rRNAs*, and CR of different Hirudinea.
