# Supplementary material for: The Complete Mitochondrial Genome of Torix tukubana (Annelida: Hirudinea: Glossiphoniidae)
Source: Genes (Basel). 2023 Feb 1;14(2):388. doi: 10.3390/genes14020388 (PMC9957428; doi:10.3390/genes14020388)
Supplement: Supplementary file 1 [file genes-14-00388-s001.zip › SupMaterial-tables1.docx]

**Table S1. Composition and skewness in PCGs, *tRNAs*, *rRNAs*, and CR of different Hirudinea**

| **Species** | **Size**  **(bp)** | **Nucleotide composition/%** | | | | | **AT-skew** | **GC-skew** |
| --- | --- | --- | --- | --- | --- | --- | --- | --- |
|  |  | **T(U)** | **C** | **A** | **G** | **A+T（U）** |  |  |
| PCGs |  |  |  |  |  |  |  |  |
| *T. tukubana* | 11097 | 39.6 | 15.2 | 33.7 | 11.4 | 73.3 | -0.080 | -0.143 |
| *A. peledina* | 11064 | 36.4 | 19.2 | 33.4 | 11.0 | 69.8 | -0.043 | -0.272 |
| *Codonobdella. sp. IK-2021* | 11066 | 39.4 | 12.9 | 35.7 | 12.0 | 75.1 | -0.049 | -0.036 |
| *E. japonica* | 11065 | 38.5 | 15.3 | 33.6 | 12.6 | 72.1 | -0.068 | -0.097 |
| *E. octoculata* | 11013 | 41.5 | 13.2 | 29.3 | 16.0 | 70.8 | -0.172 | 0.096 |
| *Erpobdella sp. JP-2021* | 11038 | 36.6 | 18.8 | 32.1 | 12.4 | 68.7 | -0.066 | -0.205 |
| *E. testacea* | 11030 | 37.8 | 15.2 | 35.1 | 12.0 | 72.9 | -0.037 | -0.118 |
| *G. concolor* | 11004 | 40.7 | 13.4 | 33.9 | 12.0 | 74.6 | -0.091 | -0.055 |
| *H. crenata* | 11002 | 43.7 | 11.7 | 32.2 | 12.5 | 75.9 | -0.152 | 0.033 |
| *H. tianmushana* | 10979 | 44.3 | 11.0 | 33.1 | 11.6 | 77.4 | -0.145 | 0.027 |
| *H. acuecueyetzin* | 11064 | 40.4 | 15.4 | 33.2 | 11.0 | 73.6 | -0.098 | -0.167 |
| *H. officinalis* | 11061 | 40.4 | 15.7 | 32.7 | 11.2 | 73.1 | -0.105 | -0.167 |
| *H. yangtzenensis* | 11089 | 39.1 | 15.5 | 34.3 | 11.1 | 73.4 | -0.065 | -0.165 |
| *H. manillensis* | 11080 | 41.5 | 12.9 | 29.6 | 16.0 | 71.1 | -0.167 | 0.107 |
| *H. medicinalis* | 11055 | 43.9 | 11.2 | 31.8 | 13.1 | 75.7 | -0.160 | 0.078 |
| *H. nipponia* | 11093 | 41.8 | 12.4 | 30.1 | 15.7 | 71.9 | -0.163 | 0.117 |
| *H. verbana* | 11043 | 44.5 | 10.8 | 32.1 | 12.6 | 76.6 | -0.162 | 0.077 |
| *O. jantseanus* | 11071 | 38.6 | 15.8 | 33.3 | 12.3 | 71.9 | -0.074 | -0.125 |
| *P. lamothei* | 11073 | 37.2 | 19.2 | 29.8 | 13.8 | 67 | -0.110 | -0.164 |
| *P. parasitica* | 11080 | 39.4 | 16.2 | 32.2 | 12.2 | 71.6 | -0.101 | -0.141 |
| *W. acranulata* | 11031 | 41.9 | 12.5 | 29.1 | 16.6 | 71 | -0.180 | 0.141 |
| *W. laevis 1* | 11007 | 42.2 | 12.6 | 29.1 | 16.2 | 71.3 | -0.184 | 0.125 |
| *W. laevis 2* | 11021 | 43.2 | 11.4 | 29.3 | 16.1 | 72.5 | -0.192 | 0.171 |
| *W. pigra* | 10989 | 42.6 | 12.2 | 29.0 | 16.3 | 71.6 | -0.190 | 0.144 |
| *Z. arugamensis* | 11055 | 41.3 | 11.6 | 36.5 | 10.7 | 77.8 | -0.062 | -0.040 |
| *tRNA* |  |  |  |  |  |  |  |  |
| *T. tukubana* | 1398 | 35.7 | 12.3 | 38.2 | 13.8 | 73.9 | 0.034 | 0.057 |
| *A. peledina* | 1404 | 33.8 | 13.8 | 39 | 13.5 | 72.8 | 0.071 | -0.011 |
| *Codonobdella. sp. IK-2021* | 1402 | 36.9 | 10.5 | 39 | 13.6 | 75.9 | 0.028 | 0.129 |
| *E. japonica* | 1396 | 34.4 | 12.3 | 37.8 | 15.5 | 72.2 | 0.047 | 0.115 |
| *E. octoculata* | 1405 | 38.4 | 10.2 | 36.7 | 14.7 | 75.1 | -0.023 | 0.181 |
| *Erpobdella sp. JP-2021* | 1325 | 33.4 | 14.4 | 36.4 | 15.8 | 69.8 | 0.043 | 0.046 |
| *E. testacea* | 1284 | 34.5 | 11.8 | 38.6 | 15.0 | 73.1 | 0.056 | 0.119 |
| *G. concolor* | 1323 | 35 | 11.5 | 39.9 | 13.6 | 74.9 | 0.065 | 0.084 |
| *H. crenata* | 1364 | 39.4 | 8.4 | 40.5 | 11.7 | 79.9 | 0.014 | 0.164 |
| *H. tianmushana* | 1346 | 38.6 | 8.8 | 41.3 | 11.3 | 79.9 | 0.034 | 0.124 |
| *H. acuecueyetzin* | 1384 | 35.5 | 12 | 38.9 | 13.5 | 74.4 | 0.046 | 0.059 |
| *H. officinalis* | 1393 | 35.7 | 12.1 | 38.3 | 13.9 | 74.0 | 0.035 | 0.069 |
| *H. yangtzenensis* | 1407 | 35.1 | 12.9 | 38.1 | 13.9 | 73.2 | 0.041 | 0.037 |
| *H. manillensis* | 1423 | 37.5 | 10.5 | 36.7 | 15.3 | 74.2 | -0.011 | 0.186 |
| *H. medicinalis* | 1333 | 38.8 | 9.2 | 39.1 | 12.9 | 77.9 | 0.004 | 0.167 |
| *H. nipponia* | 1401 | 37.3 | 10.3 | 37.3 | 15.2 | 74.6 | 0.000 | 0.192 |
| *H. verbana* | 1335 | 38.8 | 9.1 | 39 | 13.1 | 77.8 | 0.003 | 0.180 |
| *O. jantseanus* | 1442 | 35.2 | 13.2 | 36.5 | 15.1 | 71.7 | 0.018 | 0.067 |
| *P. lamothei* | 1460 | 33.6 | 13.4 | 38.3 | 14.8 | 71.9 | 0.065 | 0.050 |
| *P. parasitica* | 1461 | 33.6 | 13.2 | 38.5 | 14.6 | 72.1 | 0.068 | 0.050 |
| *W. acranulata* | 1394 | 38.7 | 10.5 | 35.9 | 14.8 | 74.6 | -0.038 | 0.170 |
| *W. laevis 1* | 1415 | 39.3 | 9.7 | 36.5 | 14.5 | 75.8 | -0.037 | 0.198 |
| *W. laevis 2* | 1360 | 38.2 | 9.5 | 37.4 | 15 | 75.6 | -0.011 | 0.224 |
| *W. pigra* | 1377 | 38.3 | 9.7 | 37.1 | 14.9 | 75.4 | -0.016 | 0.211 |
| *Z. arugamensis* | 1412 | 36.4 | 10.4 | 38.7 | 14.5 | 75.1 | 0.031 | 0.165 |
| *rRNA* |  |  |  |  |  |  |  |  |
| *T. tukubana* | 1895 | 33.9 | 12.2 | 40.6 | 13.3 | 74.5 | 0.090 | 0.043 |
| *A. peledina* | 1888 | 32.4 | 14.4 | 40.5 | 12.7 | 72.9 | 0.111 | -0.063 |
| *Codonobdella. sp. IK-2021* | 1839 | 36.5 | 10.8 | 39.9 | 12.8 | 76.4 | 0.045 | 0.085 |
| *E. japonica* | 1908 | 30.6 | 14.4 | 40.7 | 14.4 | 71.3 | 0.142 | 0.000 |
| *E. octoculata* | 1922 | 36.3 | 10.8 | 36.6 | 16.3 | 72.9 | 0.004 | 0.203 |
| *Erpobdella sp. JP-2021* | 1920 | 32.8 | 14.3 | 39.1 | 13.9 | 71.9 | 0.088 | -0.014 |
| *E. testacea* | 1950 | 31.3 | 13 | 42.2 | 13.5 | 73.5 | 0.148 | 0.019 |
| *G. concolor* | 1887 | 32.4 | 12.1 | 42.0 | 13.5 | 74.4 | 0.129 | 0.055 |
| *H. crenata* | 1853 | 38.6 | 9.0 | 39.8 | 12.6 | 78.4 | 0.015 | 0.167 |
| *H. tianmushana* | 1856 | 37.3 | 9.5 | 40.6 | 12.6 | 77.9 | 0.042 | 0.140 |
| *H. acuecueyetzin* | 1902 | 34.8 | 12.2 | 40.1 | 12.9 | 74.9 | 0.071 | 0.028 |
| *H. officinalis* | 1914 | 35.1 | 12.1 | 39.7 | 13.1 | 74.8 | 0.061 | 0.040 |
| *H. yangtzenensis* | 1903 | 33.1 | 12.7 | 40.8 | 13.4 | 73.9 | 0.104 | 0.027 |
| *H. manillensis* | 1851 | 37.7 | 10.0 | 37.5 | 14.7 | 75.2 | -0.003 | 0.190 |
| *H. medicinalis* | 1879 | 37.8 | 9.9 | 37.5 | 14.8 | 75.3 | -0.004 | 0.198 |
| *H. nipponia* | 1877 | 37.9 | 10.4 | 36.9 | 14.8 | 74.8 | -0.013 | 0.175 |
| *H. verbana* | 1882 | 37 | 10.4 | 37.8 | 14.8 | 74.8 | 0.011 | 0.175 |
| *O. jantseanus* | 1918 | 32.5 | 12.8 | 40.4 | 14.2 | 72.9 | 0.108 | 0.052 |
| *P. lamothei* | 1899 | 31.6 | 15.3 | 37.7 | 15.4 | 69.3 | 0.088 | 0.003 |
| *P. parasitica* | 1903 | 32.5 | 14.0 | 39.8 | 13.7 | 72.3 | 0.101 | -0.011 |
| *W. acranulata* | 1852 | 36.1 | 10.9 | 36.9 | 16.1 | 73.0 | 0.011 | 0.193 |
| *W. laevis 1* | 1878 | 36.3 | 10.9 | 36.6 | 16.3 | 72.9 | 0.004 | 0.199 |
| *W. laevis 2* | 1875 | 36.4 | 10.6 | 36.7 | 16.3 | 73.1 | 0.004 | 0.212 |
| *W. pigra* | 1870 | 36.1 | 11.0 | 36.5 | 16.3 | 72.6 | 0.006 | 0.194 |
| *Z. arugamensis* | 1893 | 37.4 | 9.8 | 40.8 | 11.9 | 78.2 | 0.043 | 0.097 |
| CR |  |  |  |  |  |  |  |  |
| *T. tukubana* | 402 | 31.8 | 14.2 | 41.3 | 12.7 | 73.1 | 0.130 | -0.056 |
| *A. peledina* | 444 | 36.3 | 15.3 | 42.6 | 5.9 | 78.9 | 0.080 | -0.443 |
| *Codonobdella. sp. IK-2021* | 142 | 47.2 | 7.7 | 35.2 | 9.9 | 82.4 | -0.146 | 0.125 |
| *E. japonica* | 337 | 35.6 | 13.4 | 41.2 | 9.8 | 76.8 | 0.073 | -0.155 |
| *E. octoculata* | 96 | 41.7 | 6.3 | 38.5 | 13.5 | 80.2 | -0.040 | 0.364 |
| *Erpobdella sp. JP-2021* | 1040 | 38.7 | 12 | 37.8 | 11.5 | 76.5 | -0.012 | -0.021 |
| *H. crenata* | 362 | 38.7 | 8.3 | 43.6 | 9.4 | 82.3 | 0.060 | 0.062 |
| *H. tianmushana* | 303 | 38.6 | 8.9 | 44.6 | 7.9 | 83.2 | 0.072 | -0.060 |
| *H. acuecueyetzin* | 646 | 33.1 | 19.3 | 38.1 | 9.4 | 71.2 | 0.070 | -0.345 |
| *H. officinalis* | 490 | 30.4 | 20.8 | 38.8 | 10.0 | 69.2 | 0.121 | -0.351 |
| *H. yangtzenensis* | 614 | 31.4 | 17.9 | 28.5 | 22.1 | 59.9 | -0.048 | 0.105 |
| *H. manillensis* | 77 | 31.2 | 7.8 | 41.6 | 19.5 | 72.8 | 0.143 | 0.429 |
| *H. medicinalis* | 302 | 47.7 | 5.3 | 40.4 | 6.6 | 88.1 | -0.083 | 0.109 |
| *H. nipponia* | 83 | 36.1 | 14.5 | 43.4 | 6.0 | 79.5 | 0.092 | -0.415 |
| *H. verbana* | 262 | 46.9 | 5.3 | 42.7 | 5.0 | 89.6 | -0.047 | -0.029 |
| *O. jantseanus* | 299 | 35.1 | 8.7 | 49.8 | 6.4 | 84.9 | 0.173 | -0.152 |
| *P. lamothei* | 636 | 39.2 | 12.7 | 36.5 | 11.6 | 75.7 | -0.036 | -0.045 |
| *P. parasitica* | 398 | 30.7 | 14.8 | 39.4 | 15.1 | 70.1 | 0.124 | 0.010 |
| *W. acranulata* | 87 | 32.2 | 23.0 | 31 | 13.8 | 63.2 | -0.019 | -0.250 |
| *W. laevis 1* | 78 | 23.1 | 12.8 | 42.3 | 21.8 | 65.4 | 0.294 | 0.260 |
| *W. laevis 2* | 107 | 36.4 | 9.3 | 42.1 | 12.1 | 78.5 | 0.073 | 0.131 |
| *W. pigra* | 80 | 30.0 | 12.5 | 48.8 | 8.8 | 78.8 | 0.239 | -0.174 |
| *Z. arugamensis* | 1670 | 72.5 | 4.0 | 20.4 | 3.2 | 92.9 | -0.561 | -0.111 |
