# Supplementary figures and images for: The Complete Mitochondrial Genome of Torix tukubana (Annelida: Hirudinea: Glossiphoniidae)
Source: Genes (Basel). 2023 Feb 1;14(2):388. doi: 10.3390/genes14020388 (PMC9957428; doi:10.3390/genes14020388)

**
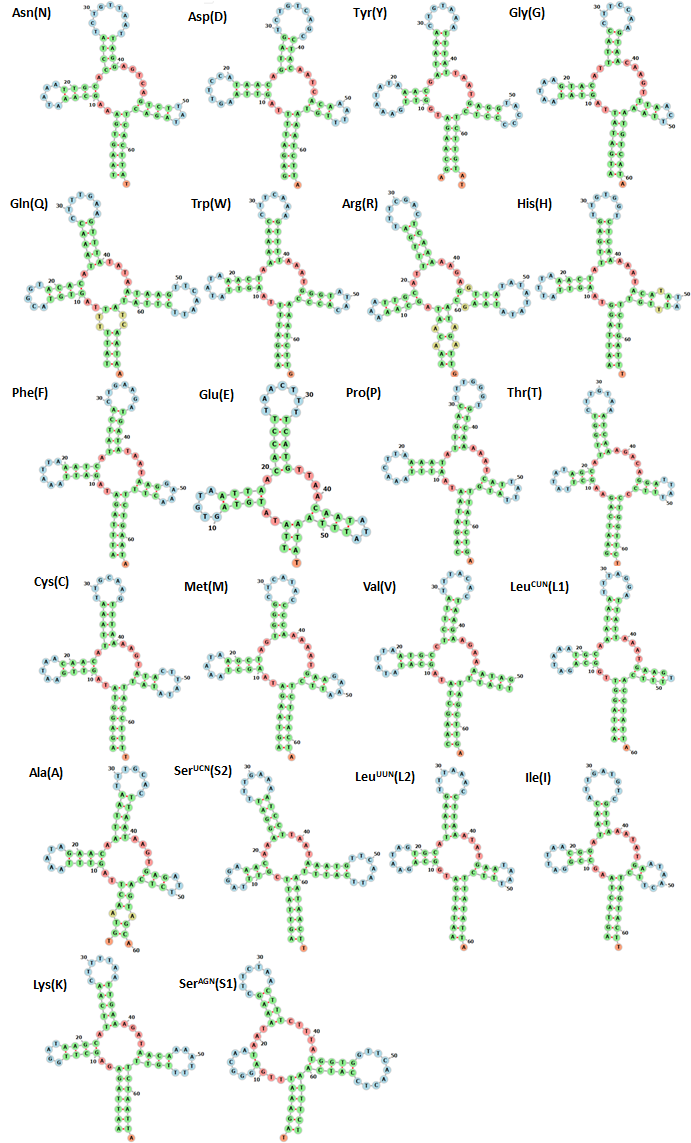
**

**Figure S1**. Predicted secondary structures of the 22 *tRNA* genes of the *T. tukubana* mitogenome.

Supplement: Supplementary file 1 [file genes-14-00388-s001.zip › SupMaterial-figs1.docx]
